# PBP-2 Negative Methicillin Resistant* Staphylococcus schleiferi* Bacteremia from a Prostate Abscess: An Unusual Occurrence

**DOI:** 10.1155/2016/8979656

**Published:** 2016-03-22

**Authors:** Chandni Merchant, Daphne-Dominique Villanueva, Ishan Lalani, Margaret Eng, Yong Kang

**Affiliations:** ^1^Department of Internal Medicine, Monmouth Medical Center, Long Branch, NJ 07740, USA; ^2^Department of Pathology, Monmouth Medical Center, Long Branch, NJ 07740, USA

## Abstract

*Staphylococcus schleiferi* subsp.* schleiferi* is a coagulase-negative* Staphylococcus* which has been described as a pathogen responsible for various nosocomial infections including bacteremia, brain abscess, and infection of intravenous pacemakers. Recently, such bacteria have been described to be found typically on skin and mucosal surfaces. It is also believed to be a part of the preaxillary human flora and more frequently found in men. It is very similar in its pathogenicity with* Staphylococcus aureus* group and expresses a fibronectin binding protein. Literature on this pathogen reveals that it commonly causes otitis among dogs because of its location in the auditory meatus of canines. Also, it has strong association with pyoderma in dogs. The prime concern with this organism is the antibiotic resistance and relapse even after appropriate treatment. Very rarely, if any, cases have been reported about prostatic abscess (PA) with this microbe. Our patient had a history of recurrent UTIs and subsequent PA resulting in* S. schleiferi* bacteremia in contrast to gram negative bacteremia commonly associated with UTI. This organism was found to be resistant to methicillin, in spite of being negative for PBP2, which is a rare phenomenon and needs further studies.

## 1. Introduction

Urinary tract infections are a less common phenomenon in male patients. Occasionally, we come across cases of prostatic abscess in adult males which can result in bacteremia caused by common urinary pathogens. We present a case of PA in an uncontrolled diabetic patient with* S. schleiferi* bacteremia, which is an uncommon organism in humans. It is more frequently found in men. Immunosuppression is a risk factor, especially malignant neoplasms, for infection with this organism. It can be nosocomial or community acquired.

## 2. Case Presentation 

This is a case of a 49-year-old Caucasian male with past medical history significant for diabetes mellitus, hypertension, benign prostatic hyperplasia, and recurrent folliculitis who presented to the Emergency Department (ED) complaining of fever and chills of three-day duration. The patient was apparently well until two months priorly; the patient sought consult at the ED for one-week history of gross hematuria, increased frequency, dysuria, and suprapubic and perineal discomfort. He was diagnosed to have acute prostatitis and was sent home on a 2-week course of ciprofloxacin 500 mg BID. In the interim, the patient reported occasional episodes of gross hematuria and consulted his primary care physician (PCP) who prescribed an additional 1-week course of the same antibiotic. Three days prior to admission, the patient developed fever (*T*_max_ 102.7 F) and chills associated with nonbilious vomiting, loose bowel movement, poor appetite, and generalized body weakness. The patient also reported dysuria, intermittency, and hesitancy. He had no sick contacts and no history of recent travel. The patient works as a landscaper and lives at home with his wife. He used to occasionally drink alcohol (stopped at age 46) and is a former smoker (stopped at age 21) and he denies illicit drug use.

At the ED, the patient had low-grade fever (100.7 F). He is obese and on further inspection had very large tattoos on his back and arms. On digital rectal examination, external haemorrhoids were seen and the prostate was noted to be enlarged and nontender. Blood work revealed leukocytosis (WBC 14.1 K/mm^3^) with bandemia (20%). Urinalysis was positive for 30 mg/dL urinary protein, small amount of blood, 15 mg/dL of ketones, moderate amount of leukocyte esterase, too numerous to count WBC, and 1+ bacteria. At the ED, the patient was managed as a case of complicated UTI for which he received one dose of IV levofloxacin 750 mg. On admission to the medical floor, diagnosis was fever and leukocytosis possibly due to prostatitis/abscess ruling out* Clostridium difficile* diarrhea. Antibiotic was shifted to IV cefepime 1 g daily. Urine, stool, and blood cultures were sent.* Clostridium difficile* toxin PCR returned positive and two sets of blood culture grew gram positive cocci in clusters. In addition, CT scan of the abdomen and pelvis showed lobulated fluid density within the prostate gland extending to the right superior aspect. The working diagnosis at this time was prostate abscess,* methicillin-sensitive S. aureus (MSSA)* bacteremia, and* Clostridium difficile* diarrhea. Antibiotic was shifted to IV vancomycin 1 g q12 and PO vancomycin 125 mg q6. Infectious disease (ID) and genitourinary (GU) specialists were consulted. IV vancomycin was changed to IV cefazolin 2 g q8 for* S. aureus* subsp.* aureus* bacteremia likely from prostate abscess. The* S. aureus* subsp.* aureus* bacteremia was found to be negative for PBP-2, an immune-chromatographic assay, Alere*™* PBP2a SA Culture Colony Test. On his fourth hospital day, the patient underwent cystoscopy with transurethral resection of the prostate (TURP), deroofing of the abscess, and foley catheter insertion. The specimen that consisted of multiple fragments of gray-tan soft tissue measuring 3.5 × 1.7 × 0.3 cm in aggregate was submitted for microscopic examination. It showed an organizing abscess. As* S. aureus* prostatitis is not a common condition and may be related to haematogenous spread, a transthoracic echocardiogram (TTE) was done and it revealed normal left ventricular systolic function with no evidence of infective endocarditis. Blood culture was noted to grow coagulase negative* S. schleiferi* subsp.* schleiferi*, using the machine BacTec 9240, Maryland, USA. It was found to be resistant to cefazolin; hence antibiotic was shifted back from IV cefazolin to IV vancomycin 1 g q12. Antibiotic susceptibility results for* S. schleiferi* demonstrated sensitivity to clindamycin, gentamycin, levofloxacin, tetracycline, sulfamethoxazole/trimethoprim, and vancomycin. The technique used to detect antibiotic susceptibility was the FDA-approved Microscan, Illinois, USA.

Working diagnosis at this time was* S. schleiferi* bacteremia from prostate abscess status after cystoscopy, TURP with deroofing of abscess, and* Clostridium difficile* diarrhea. WBC and fever were noted to trend down and the patient had voiding trial without difficulty. On his 8th hospital day, the patient was discharged on sulfamethoxazole/trimethoprim BID for 21 days and vancomycin 125 mg q6 to complete 14 days. The patient came back to the ED one week after discharge complaining of two-day history of diarrhea and hematuria. He was readmitted for* Clostridium difficile* relapse and started on treatment with oral vancomycin 125 mg every 6 hours. He was reassured that hematuria is expected after a genitourinary procedure and may be seen intermittently for 4 to 6 weeks. He was then discharged to complete a course of oral vancomycin with a stable follow-up course with his PCP and Urologist.

## 3. Discussion

*Staphylococcus schleiferi* subsp.* schleiferi* is coagulase-negative* Staphylococcus* first described in April 1988. It has been implicated as the causative pathogen in a range of nosocomial infections like bacteremia, brain abscess, pacemaker- and other intravenous-device-related infections, and infections of the urinary tract, orthopedic implants, and surgical wounds [1].* S. schleiferi* subsp.* coagulans* is coagulase positive but instead* S. schleiferi* subsp.* schleiferi* is coagulase negative. It is recently discovered that skin and multiple mucosal surfaces are the typical sites where the bacteria can be found. It is also believed to be a part of the preaxillary human flora. It causes otitis in dogs due to its location in the auditory meatus of dogs [2]. Antibiotic resistance has resulted in increased pathogenicity of this organism. It was newly found to be pathogenic to humans and other mammals. Infection tends to relapse even after antibiotic treatment. In a European study, it was found that, in 0.7% of the studied patients' urine samples,* S. schleiferi* was found. This study comprised 4,905 patients in total, whose urine samples were analyzed [3]. All of these 3 cases, a 64-year-old female, a 68-year-old male, and a 3-month-old male, with colony counts of 468,000 cfu/mL, 324,000 cfu/mL, and 764,000 cfu/mL, respectively, were inpatients. This fact leads us to the hypothesis that, among coagulase-negative staphylococci,* S. schleiferi*, a newly described species of coagulase-negative staphylococci not previously reported as a uropathogen, may also cause hospital-acquired urinary tract infection. After meticulous review of the literature, we did not come across any case of prostate abscess being caused by coagulase-negative* S. schleiferi*.

This case illustrates an example of thinking out of the norms and delivering precise treatment. Our patient had long-standing history of urinary tract infection and was found to have bacteremia during the course of his hospital stay. The blood cultures were positive for a growth of coagulase-negative* S. schleiferi*. Bacteremia after urinary tract infection is mostly associated with gram negative organisms.* S. schleiferi* is differentiated from* S. aureus* by production of a heat stable nuclease. Other identification tests used are novobiocin susceptibility, coagulase negativity, and fibrinogen affinity factor. On detailed workup for a possible source of Staphylococcal sepsis, the only source was found to be a prostate abscess. The virulence factors of these bacteria are the production of beta-hemolysin, lipase, and esterase [4]. The mechanisms by which* S. schleiferi* cause these infections are unknown, but there is a degree of similarity between the spectrum of infections caused by this organism and those associated with* S. aureus*. Isolation of* S. schleiferi* from cultures of prosthetic material suggests the presence of one or more bacterial cell surface-expressed adhesins with a host protein specificity similar to that of* S. aureus*.* S. schleiferi* has been reported to bind fibrinogen, but adherence to fibronectin and the identification of cell wall-associated adhesins have not previously been described for this organism [1]. In our patient, the isolated bacteria from blood cultures were found to be negative for PBP-2 (penicillin binding protein-2) which is encoded by MecA gene, which is the mechanism for resistance to methicillin and other penicillin or beta-lactam antibiotics [5]. Surprisingly, in this case, in spite of absence of PBP-2, the susceptibility results revealed that the* S. schleiferi* bacteria were resistant to cefazolin and oxacillin. A review article in 2014 describes the emergence of mecC MRSA, cases of which have only been reported in Europe till date, and they pose a challenge in diagnosis where only mecA or PBP2a/2′ are used for MRSA detection [6]. In the future, laboratories will need to start using the universal mec gene primers for PCR amplification of both the mecA and mecC gene. In a recent study in 2015 which studied 217 isolates from canine specimens from mid-Atlantic and regions across the USA, it was found that of the mid-Atlantic isolates 62% (72 of 116) were methicillin resistant (MR) and 16% (18 of 116) were multidrug resistant (MDR). Of the isolates from the other geographic regions, 73% (74 of 101) were MR and 24% (24 of 101) were MDR [7]. This reflects the prevalence of antibiotic resistance pattern in this organism across USA. It has been recently studied that methicillin resistance is common with* S. schleiferi* isolates in veterinarian studies. Fluoroquinolone resistance is also noted in methicillin resistant* S. schleiferi (MRSS)* [7]. However, in our hospital, the antibiogram for* S. schleiferi* reveals 100% sensitivity to fluoroquinolones which included ciprofloxacin, levofloxacin, and moxifloxacin.

In the preantibiotic decades, prostate abscesses (PA) frequently had a dramatic presentation and were usually caused by* Neisseria gonorrhoeae*. In the present times, PA may be difficult to differentiate from prostatitis and other diseases of the lower urogenital tract. The organisms most frequently isolated from PA are* Escherichia coli* and other gram negative bacilli; other isolates include* Staphylococcus* species and an increasing range of various bacteria and fungi. PA due to* Staphylococcus* species may also occur in neonates [8]. The patient denied having any pets or exposure to dogs in his community. Our patient was a poorly controlled diabetic, which could lead to immune suppression. He did not have any history of recent hospitalizations. Antibiotics were changed multiple times in his case. This happens in situations when rare microorganisms play a role in disease causation, and one has to be prudent about selection of the right antimicrobial agents. With advent of antibiotic overuse, we should be ready for infections caused by newer unusual pathogens and use the latest tests to detect serious infections like mecA negative MRSA or MRSS.
